# Impact of telomere length on autoimmune thyroid disease in Europeans: insights from Mendelian randomization

**DOI:** 10.1016/j.clinsp.2025.100765

**Published:** 2025-09-03

**Authors:** Gang Hu, Yunfeng Yu, Yuman Yin, Xinyu Yang, Rong Yu, Qin Xiang

**Affiliations:** a School of Traditional Chinese Medicine, Hunan University of Chinese Medicine, Hunan, China; b The First Hospital of Hunan University of Chinese Medicine, Hunan, China

**Keywords:** Telomere length, Autoimmune thyroid disease, Autoimmune thyroiditis, Graves disease, Mendelian randomization

## Abstract

**Objective:**

This study aimed to investigate the causal relationship between Telomere Length (TL) and Autoimmune Thyroid Disease (AITD) in Europeans using Mendelian Randomization (MR).

**Methods:**

Single nucleotide polymorphisms associated with TL and AITD were obtained from genome-wide association studies. MR analysis was conducted using inverse variance weighted as the primary method. Cochran's *Q* test and leave-one-out sensitivity analysis were employed to assess heterogeneity and robustness, respectively.

**Results:**

TL was significantly associated with reduced genetic susceptibility to Graves' Disease (GD) in Europeans (Odds Ratio [OR = 0.776], 95 % Confidence Interval [95 %CI 0.625–0.964]; *p* = 0.022). However, no association was found between TL and Autoimmune Thyroiditis (AIT) (OR = 1.474, 95 % CI 0.870–2.497; *p* = 0.149). Cochran's *Q* test and leave-one-out sensitivity analysis confirmed that the findings were free of heterogeneity and robust.

**Conclusion:**

MR analysis revealed that TL shortening is associated with increased genetic susceptibility to GD in Europeans, but no such association was observed for AIT. Further research is needed to elucidate the mechanisms through which TL influences AITD.

## Highlights

• Telomere length is associated with Graves' disease risk in Europeans.
• Telomere length is not associated with autoimmune thyroiditis risk in Europeans.
• The impact of telomere length on autoimmune thyroid disease in Europeans is unequal.

## Introduction

Telomeres are protein-DNA complexes located at the ends of eukaryotic linear chromosomes that prevent chromosome degradation from nucleolytic attack, thereby maintaining genome stability and integrity.^1^^,^^2^ Since Telomere Length (TL) progressively decreases with each cell cycle division,^3^ it is considered a reliable marker of cellular senescence.^4^ TL shortening induces DNA damage, which subsequently triggers cellular apoptosis and senescence, ultimately contributing to aging-related diseases.^5^ Currently, leukocyte TL is the primary indicator used by clinical researchers, reflecting the relative TL across most tissues.^6^ Studies have shown that shortening of TL is associated with an increased risk of various autoimmune diseases and cancers.^7^^,^^8^ Investigating the role and mechanism of TL in disease susceptibility may enhance understanding of these processes and facilitate the development of therapeutic strategies.

Autoimmune Thyroid Disease (AITD) is an autoimmune disorder characterized by acquired thyroid dysfunction.^9^ The two most common subtypes are Autoimmune Thyroiditis (AIT) and Graves' Disease (GD). AIT is the main AIT of hypothyroidism, with Thyroid Peroxidase Antibody (TPOAb) and Thyroglobulin Antibody (TGAb) serving as specific markers.^10^ In contrast, GD is the leading cause of hyperthyroidism, characterized by Thyrotropin Receptor Antibody (TRAb) targeting Thyroid Stimulating Hormone (TSH) receptors.^11^ The incidence of AITD is reported to be 2 % to 5 % in Western countries.^12^ Beyond being a prevalent autoimmune disorder, AITD is also a significant risk factor for thyroid cancer,^13^ making it a focal point of research in the current immunological research. Previous studies have linked shortened leukocyte TL with an increased risk of GD in East Asians.^14^ However, the relationship between TL and AIT, as well as the role of TL in the genetic susceptibility of Europeans to AITD, remains unclear. Therefore, further research is needed to investigate the causal relationship between TL and AITD in European populations.

Mendelian Randomization (MR) is an analytical approach used to assess the causal relationship between exposures and outcomes.^15^ By treating genetic variation as an intermediate pathway, it is less susceptible to confounding variables and reverse causation.^15^ This study employed MR to evaluate the effect of TL on genetic susceptibility to AITD in Europeans.

## Materials and methods

### Study design

**Fig. 1:**
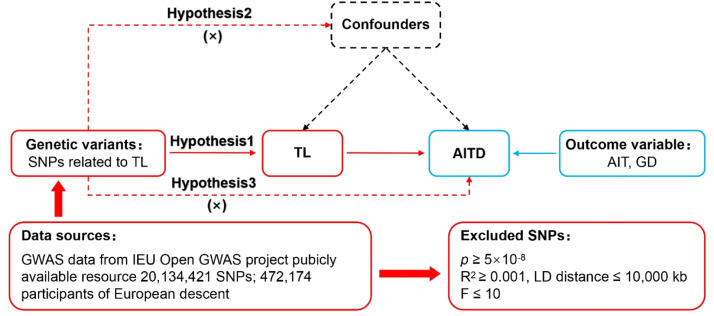
MR design for TL on genetic susceptibility to AITD. MR, Mendelian Randomization; TL, Telomere Length; AITD, Autoimmune Thyroid Disease; AIT, Autoimmune Thyroiditis; GD, Graves' Disease.

MR was based on three fundamental assumptions: association, independence, and exclusivity.^16^ The association assumption requires that Single Nucleotide Polymorphisms (SNPs) be strongly associated with the exposure. The independence assumption stipulates that SNPs were independent of confounding variables. The exclusivity assumption mandates that SNPs act on the outcome only through the exposure and not through other pathways. The MR design for TL and AITD is illustrated in Fig. 1.

### Data sources

Table 1 summarizes the data sources for exposure and outcomes. The dataset for TL, numbered ieu-b-4879, was obtained from IEU Open GWAS project (gwas.mrcieu.ac.uk/), which included data from 472,174 Europeans.^17^ Leukocyte TL was measured using multiplexed quantitative polymerase chain reaction in a UK biobank cohort of individuals aged 45 to 69 years, collected between 2006 and 2010. This method assessed telomere repeat copy number to single-copy gene ratios, applying stringent quality control and stability evaluations.

**Table 1:** Details of the GWAS studies included in the Mendelian randomization.

Year	Trait	GWAS ID	Population	Sample size	Web source
2021	TL	ieu-b-4879	European	472,174	gwas.mrcieu.ac.uk/
2023	AIT	finngen_R10_E4_THYROIDITAUTOIM	European	350,256	www.finngen.fi/en
2023	GD	finngen_R10_E4_GRAVES_STRICT	European	412,181	www.finngen.fi/en

TL, Telomere Length; AIT, Autoimmune Thyroiditis; GD, Graves' Disease.

The datasets for AIT and GD, identified as finngen_R10_E4_THYROIDITAUTOIM and finngen_R10_E4_GRAVES_STRICT were obtained from FinnGen (www.finngen.fi/fi).^18^ FinnGen compiles samples from the nationwide network of Finnish biobanks and digital healthcare data from the national health registry and combines pseudonymized registry data with the minimum phenotype dataset provided by the Finnish biobanks. FinnGen defines AIT as an inflammatory disease of the thyroid gland caused by autoimmune responses, leading to lymphocytic infiltration of the gland. It is characterized by circulating thyroid antigen-specific T-cells and thyroid autoantibodies. Clinical manifestations range from hypothyroidism to thyrotoxicosis depending on the type of AIT. In addition, FinnGen defines GD as an autoimmune disorder that causes hyperactivity of the thyroid gland (hyperthyroidism), which is caused by an abnormal immune response that stimulates excessive thyroid hormone production by the thyroid gland. FinnGen provided Genome-Wide Association Study (GWAS) data for AIT from 350,256 Europeans, including 539 in the experimental group and 349,717 in the control group. Similarly, GWAS data for GD included 412,181 Europeans, with 3176 in the experimental group and 409,005 in the control group. As these datasets originated from publicly available databases, no additional ethical approval was required for this study.

### Genetic instrumental variable selection

Genetic Instrumental Variables (IVs) were selected through a systematic process. SNPs strongly associated with TL were first identified using a threshold of *p* 〈 5 × 10^–8^. To ensure independence among variables, SNPs with *R^2^* < 0.001 and a genomic distance of 10,000 kb were selected. SNPs with a strong correlation were included based on a threshold of *F 〉* 10, calculated as *F* = [*R*^2^/(1-*R*^2^)]*[(*N*-*K*-1)/*K*], where *R^2^* represents the cumulative explained variance of the selected IVs with the exposure, *N* denotes the GWAS sample size, and *K* represents the number of paired samples. To maintain the assumptions of independence and exclusivity, the authors searched SNPs' meanings using PhenoScanner, PubMed, and the Web of Science, and excluding those with confounding variables. Mismatched SNPs were also excluded based on the effect of allele frequency when adjusting for allele orientation between exposure and outcome. Finally, the MR-pleiotropy RESidual Sum and Outlier method were applied to detect and exclude outlier SNPs (*p* < 0.05), ensuring the accuracy of causal inference.

### Data analysis

This study followed the Strengthening the Reporting of Observational Studies in Epidemiology Using Mendelian Randomization (STROBE-MR) guidelines.^19^ The “TwoSampleMR (0.5.7)” package in *R* 4.3.1 was used to perform the two-sample MR analysis. The Inverse Variance Weighted (IVW) was set as the primary assessment tool, which enabled unbiased causal analysis without pleiotropy.^20^ The weighted median and MR-Egger methods were used as secondary assessment tools; the former is less sensitive to errors and outliers, whereas the latter provides effective causal analysis in the presence of pleiotropy. The MR Egger intercept was applied to assess horizontal pleiotropy, with *p* ≥ 0.05 indicating no significant horizontal pleiotropy, thereby satisfying the exclusivity assumption. Cochran's *Q* test was employed to evaluate heterogeneity, with *p* ≥ 0.05 indicating no significant heterogeneity. Leave-one-out sensitivity analysis was performed to assess the robustness of MR results, with consistent effects across all analyses indicating robust findings.

## Results

### Genetic instrumental variable

The TL datasets included 472,174 European participants and 37,529 closely related SNPs. After applying the association, independence, and exclusivity tests, 140 TL-related SNPs were selected (Supplementary Table S1). After excluding mismatched and outlier SNPs, 128 SNPs were retained for the MR analyses of TL with AIT and GD (Supplementary Tables S2 and S3).

### Two-sample MR analysis

#### Impact of TL on AIT

**Fig. 2:**
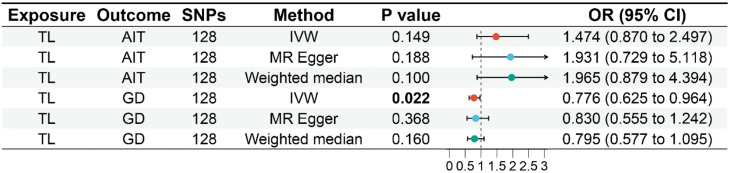
Forest plot of MR analysis for TL on genetic susceptibility to AITD. MR, Mendelian Randomization; TL, Telomere Length; AITD, Autoimmune Thyroid Disease; AIT, Autoimmune Thyroiditis; GD, Graves' Disease.

**Fig. 3:**
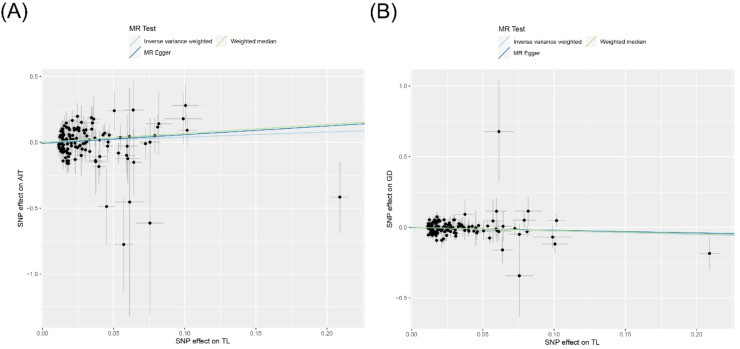
Scatter plot of MR analysis for TL on genetic susceptibility to AITD. (A) TL on AIT; (B) TL on GD. MR, Mendelian Randomization; TL, Telomere Length; AITD, Autoimmune Thyroid Disease; AIT, Autoimmune Thyroiditis; GD, Graves' Disease.

All three analytical methods indicated no significant association between TL and genetic susceptibility to AIT in Europeans: IVW (Odds Ratio [OR = 1.474], 95 % Confidence Interval [95 % CI 0.870–2.497], *p* = 0.149), MR Egger (OR = 1.931, 95 % CI 0.729–5.118, *p* = 0.188), and weighted median (OR = 1.965, 95 % CI 0.879–4.394, *p* = 0.100) (Fig. 2, Fig. 3). The MR Egger intercept demonstrated the absence of horizontal pleiotropy (*p* = 0.519) (Supplementary Table S4).

#### Impact of TL on GD

The IVW analysis suggested an association between TL and reduced genetic susceptibility to GD in Europeans (OR = 0.776, 95 % CI 0.625–0.964, *p* = 0.022), while MR Egger (OR = 0.830, 95 % CI 0.555–1.242, *p* = 0.368) and weighted median (OR = 0.795, 95 % CI 0.577–1.095, *p* = 0.160) did not support this association (Fig. 2, Fig. 3). The MR Egger intercept indicated the absence of horizontal pleiotropy (*p* = 0.695) (Supplementary Table S4).

### Heterogeneity and sensitivity analysis

**Fig. 4:**
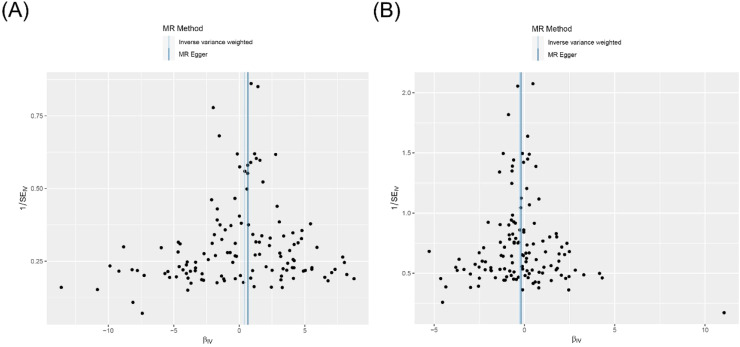
Funnel plot of heterogeneity analysis for TL on genetic susceptibility to AITD. (A) TL on AIT; (B) TL on GD. MR, Mendelian Randomization; TL, Telomere Length; AITD, Autoimmune Thyroid Disease; AIT, Autoimmune Thyroiditis; GD, Graves' Disease.

**Fig. 5:**
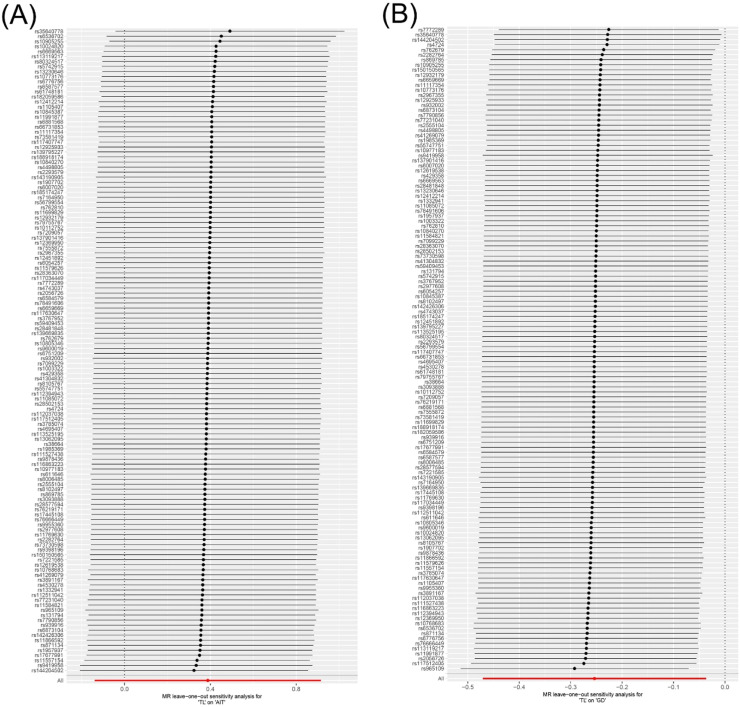
Leave-one-out sensitive analysis for TL on genetic susceptibility to AITD. (A) TL on AIT; (B) TL on GD. MR, Mendelian Randomization; TL, Telomere Length; AITD, Autoimmune Thyroid Disease; AIT, Autoimmune Thyroiditis; GD, Graves' Disease.

Cochran's *Q* test revealed no significant heterogeneity in the results for TL and AIT (*p* = 0.318) or TL and GD (*p* = 0.366) (Fig. 4 and Supplementary Table S5). In addition, the leave-one-out sensitivity analysis confirmed the robustness of the results (Fig. 5).

## Discussion

### Research background and findings

Telomeres, protective complexes located at the ends of eukaryotic chromosomes, play a critical role in preventing cellular apoptosis and senescence.^21^ TL is closely linked to inflammatory responses and immune system abnormalities, which are key mechanisms underlying metabolic diseases, reproductive disorders, and tumors.^22^^,^^23^ As research progresses, evidence increasingly suggests that TL shortening increases the risk of autoimmune diseases.^24^^,^^25^ AITD, a prevalent autoimmune condition and a significant risk factor for thyroid cancer,^26^^,^^27^ may have a complex relationship with TL. Although previous studies have associated TL shortening with an increased risk of GD in East Asians,^14^ the impact of TL on the risk of AITD in Europeans remains poorly understood. This MR analysis revealed that longer TL was associated with reduced genetic susceptibility to GD in Europeans, but not such association was found for AIT. These findings showed no evidence of horizontal pleiotropy or heterogeneity and were confirmed to be robust.

### Relationship between TL and GD

The present findings revealed an association between longer TL and a lower risk of GD, similar to previous reports. Genetic research based on Japan Biobank-1 a significant relationship between the TL shortening and an increased risk of GD in East Asians.^14^ Similarly, a study on European populations demonstrated that TL lengthening acts as a protective factor against autoimmune hyperthyroidism (OR = 0.49, 95 % CI 0.34–0.72, *p* = 2.83 × 10^–4^).^28^ These studies highlight the potential link between TL and GD in East Asians and autoimmune hyperthyroidism in Europeans. However, differences in race and disease scope limit the ability of these studies to fully explain the effect of TL on genetic susceptibility to GD in Europeans. Furthermore, aside from the aforementioned studies, no additional direct evidence is available, indicating a significant gap in research regarding the relationship between TL and GD.

Numerous studies have confirmed the association between TL and autoimmune diseases, providing indirect evidence for a potential correlation between TL and GD. For instance, TL shortening has been linked to rheumatoid arthritis, possibly mediated by the susceptibility gene HLA-DRB1×04. A clinical study in Mexico reported that patients with early-stage rheumatoid arthritis had significantly shorter TL than healthy individuals.^25^ Similarly, a study in the United States found that the TL in CD4+ and CD8+ *T*-cell subsets was significantly reduced in patients with rheumatoid arthritis compared with healthy controls.^8^ Another study in the United States demonstrated that HLA-DRB1×04 contributes to excessive telomere reduction in CD4^+^ *T*-cells, promoting senescence and accumulation of autoreactive T cells in patients with rheumatoid arthritis.^29^ TL shortening has also been implicated in the development and prognosis of Sjögren's syndrome. A study in North Carolina revealed significant telomere erosion in salivary DNA from patients with primary Sjögren's syndrome compared with healthy individuals.^30^ Another study in the Netherlands observed significantly shortened TL in G stem cells from patients with primary Sjögren's syndrome accompanied by reduced cell number, impaired differentiation, and premature senescence.^31^ TL shortening has also been documented in patients with multiple sclerosis. A clinical trial in Germany found that patients with relapsing-remitting multiple sclerosis had significantly shorter average relative leukocyte TL compared with healthy controls.^32^ Another study conducted in China confirmed that both male (6.5 ± 0.3 vs. 8.4 ± 1.9) and female patients (7.0 ± 0.9 vs. 10.1 ± 2.9) with primary progressive multiple sclerosis had shorter TL compared with the general population.^33^ Furthermore, a related meta-analysis reported that TL in patients with systemic lupus erythematosus was significantly shorter than that in the controls (standardized mean difference −0.835, 95 % CI −1.291 to −0.380), and this difference remained significant across different races, sample types, and detection methods.^34^ These findings suggest that TL shortening is associated with several autoimmune diseases, including rheumatoid arthritis, Sjögren's syndrome, systemic sclerosis, and systemic lupus erythematosus. Considering the comorbidity between GD and other autoimmune diseases,^35^ a connection between TL shortening and the onset and progression of GD can reasonably be speculated.

The additional risk of GD caused by the TL shortening may be mediated by immune dysfunction, inflammatory responses, and oxidative stress. Studies have demonstrated that TL shortening induces the senescence and apoptosis of immune cells, impairs immune system function, and ultimately contributes to tissue-damaging diseases.^36^ Chronic inflammatory responses driven by immune dysfunction, a common mechanism of autoimmune diseases,^37^ may play a role in the development and progression of GD through sustained inflammatory activity mediated by TL shortening. Furthermore, research by Guan et al.^33^ identified a link between TL shortening related to oxidative stress and the severity of systemic sclerosis, observing elevated levels of the lipid peroxidation marker 8-isoPGF2α in patients with systemic sclerosis. This finding suggests that TL shortening related to oxidative stress may similarly contribute to the pathogenesis of GD. In summary, immune dysfunction, inflammatory responses, and oxidative stress represent potential mechanisms by which TL shortening could promote the onset and progression of GD.

To investigate the genetic mechanism through which TL influences GD, the authors analyzed 128 SNPs associated with TL and GD from public databases. However, no reports currently link these SNPs to GD. Understanding the mechanism and genetic loci through which TL affects GD remains an important area for future research, underscoring the need for continued investigation and exploration.

### Relationship between TL and AIT

This study did not find a causal relationship between TL and AIT, consistent with the findings of the only relevant clinical study to date. That investigation, conducted in Germany, included 18 patients with Hashimoto's thyroiditis and 70 healthy individuals. It revealed that the relative TL levels of CD4+ CD45RA+ and CD8+ CD45RA+ in patients with Hashimoto's thyroiditis were comparable to those of healthy individuals.^38^ Considering the distinct pathogenesis of AIT and GD, the differing effects of TL on the two conditions may be attributed to their specific underlying mechanisms. However, no existing literature explains the unequal impact and mechanism of TL shortening on AIT and GD, highlighting this as a critical area for future research.

Notably, Liu et al.^28^ reported findings that differ from ours, observing a significant association between TL and the risk of autoimmune hypothyroidism (OR = 0.86, 95 % CI 0.77–0.96, *p* = 7.46 × 10^–3^). These conflicting results may stem from differences in disease scope and study populations. Although autoimmune hypothyroidism and AIT share some overlap, they are distinct conditions, which could result in varying effects of TL on each disease. In addition, the dataset used by Liu et al.^28^ for autoimmune hypothyroidism and the dataset used for AIT in the present study did not completely overlap, suggesting that clinical heterogeneity may have contributed to the differences in the observed results. In view of the limited availability of evidence-based data, further clinical studies are needed to explore the causal effects of TL on autoimmune hypothyroidism and AIT, addressing these inconsistencies.

### Clinical significance and inspiration

These findings indicate that TL is associated with reduced genetic susceptibility to GD in Europeans, suggesting that TL may serve as a biomarker for predicting the risk of GD in this population. Clinicians may consider using TL as an early risk assessment tool for European individuals at high genetic risk of GD, enabling proactive monitoring of thyroid function and the implementation of early lifestyle interventions for patients with shorter TL. However, owing to the absence of a demonstrated link between TL and AIT, the utility of TL in diagnosing or predicting AIT remains uncertain and requires further investigation.

A study conducted in China identified a significant negative correlation between the relative TL of B lymphocytes and clinical symptoms (*r* = −0.613) in patients with immune-related pancytopenia, suggesting that TL shortening may be associated with the progression of autoimmune diseases. In summary, these findings highlight a potential relationship between TL and the genetic susceptibility or severity of autoimmune diseases, offering valuable insights for future research into the role of TL in disease prediction and management.

### Limitations and prospects

This MR analysis had several limitations. First, TL ‒ the exposure factor of the study ‒ was measured in serum rather than thyroid tissue, potentially reducing the specificity of the results. Second, since both the TL and AITD datasets were derived from European populations, this study only addressed the role of TL in the risk of AITD among individuals of European descent. Third, owing to the lack of GWAS data for other subtypes of AITD, the analysis was limited to AIT and GD, which may restrict the generalizability of the results. Fourth, unrecognized confounding variables between TL and AITD could have introduced bias. Fifth, since the included datasets did not exclude patients with other comorbidities, such as diabetes or hypertension, these conditions may have influenced the objectivity and accuracy of the results. To address these limitations, further human genome research should aim to generate richer and more diverse datasets to support MR analyses across different populations. In addition, multi-center, large-sample, and stratified clinical trials are needed to minimize the influence of confounding factors and to investigate the effects of TL on various AITD subtypes more comprehensively.

## Conclusion

MR analysis indicated that TL shortening is associated with increased genetic susceptibility to GD in Europeans, but no such association was observed for AIT. Further research is needed to elucidate the effects and mechanisms of TL on various subtypes of AITD.

## Ethics in publishing

This study is based on published experimental research and is not currently applicable to medical ethics.

## Funding

This study was supported by The Key Support Project of the Regional Innovation and Development Joint Fund of the National Natural Science Foundation of China (U21A20411).

## Declaration of competing interest

The authors declare no conflicts of interest.

## Data Availability

The original contributions to this study are included in the article/Supplementary Table Material. For further inquiries, please contact the corresponding author.

## Appendix Supplementary materials

Image, application 1
