# Supplementary material for: Impact of telomere length on autoimmune thyroid disease in Europeans: insights from Mendelian randomization
Source: Clinics (Sao Paulo). 2025 Sep 3;80:100765. doi: 10.1016/j.clinsp.2025.100765 (PMC12445582; doi:10.1016/j.clinsp.2025.100765)
Supplement: Supplementary file 1 [file mmc1.docx]

CLINICS-D-24-00819_Supplementary Material

**Supplementary Table S1** Characteristics of instrumental variables for TL.

|  | **SNP** | **EA** | **OA** | **Samplesize** | **SE** | **β** | **EAF** | **p-value** | **R^2^** | **F - statistic** |
| --- | --- | --- | --- | --- | --- | --- | --- | --- | --- | --- |
| **1** | rs2977608 | C | A | 472,174 | 0.00233716 | 0.0129483 | 0.743949 | 3.02E-08 | 6.39E-05 | 30.16151229 |
| **2** | rs66731853 | A | G | 472,174 | 0.00215421 | -0.0177791 | 0.317304 | 1.54E-16 | 0.000136947 | 64.67138401 |
| **3** | rs6669563 | A | G | 472,174 | 0.00202476 | 0.0182358 | 0.437768 | 2.13E-19 | 0.000163696 | 77.30552557 |
| **4** | rs3767952 | A | G | 472,174 | 0.00238826 | 0.0134472 | 0.226709 | 1.80E-08 | 6.34E-05 | 29.9387185 |
| **5** | rs41269079 | A | T | 472,174 | 0.0025499 | 0.0153617 | 0.188991 | 1.70E-09 | 7.23E-05 | 34.15914555 |
| **6** | rs139795227 | C | A | 472,174 | 0.00873247 | 0.0599379 | 0.014021 | 6.71E-12 | 9.93E-05 | 46.90542399 |
| **7** | rs7555872 | G | A | 472,174 | 0.00498734 | 0.027267 | 0.042657 | 4.57E-08 | 6.07E-05 | 28.67406 |
| **8** | rs4498805 | T | G | 472,174 | 0.00200376 | 0.0150601 | 0.546632 | 5.65E-14 | 0.000112417 | 53.08608233 |
| **9** | rs11584821 | T | C | 472,174 | 0.00263623 | -0.0306517 | 0.176208 | 3.00E-31 | 0.000272761 | 128.8252591 |
| **10** | rs11579626 | C | A | 472,174 | 0.00357752 | 0.0265113 | 0.084882 | 1.26E-13 | 0.00010919 | 51.56231395 |
| **11** | rs6587577 | G | A | 472,174 | 0.0026359 | -0.0182148 | 0.826346 | 4.84E-12 | 9.52E-05 | 44.9642253 |
| **12** | rs6659669 | T | C | 472,174 | 0.00205167 | -0.0117091 | 0.605095 | 1.15E-08 | 6.55E-05 | 30.94011399 |
| **13** | rs932002 | T | C | 472,174 | 0.00279667 | -0.0402052 | 0.150843 | 7.31E-47 | 0.000414102 | 195.608496 |
| **14** | rs2555104 | C | A | 472,174 | 0.00203498 | -0.0139717 | 0.434255 | 6.61E-12 | 9.59E-05 | 45.29350601 |
| **15** | rs56178008 | A | T | 472,174 | 0.00201464 | 0.0143739 | 0.437497 | 9.70E-13 | 0.00010169 | 48.02015435 |
| **16** | rs6751209 | C | T | 472,174 | 0.00248465 | -0.0140465 | 0.204231 | 1.57E-08 | 6.41E-05 | 30.28328655 |
| **17** | rs965109 | T | C | 472,174 | 0.00648201 | -0.101702 | 0.024338 | 1.77E-55 | 0.000491217 | 232.052833 |
| **18** | rs188918174 | T | C | 472,174 | 0.00543604 | 0.0403062 | 0.036106 | 1.22E-13 | 0.000113079 | 53.39882533 |
| **19** | rs12619538 | C | A | 472,174 | 0.00287813 | 0.0167489 | 0.141035 | 5.91E-09 | 6.80E-05 | 32.09480155 |
| **20** | rs17803849 | T | C | 472,174 | 0.00203482 | 0.0273203 | 0.405161 | 4.24E-41 | 0.000359773 | 169.9356503 |
| **21** | rs869785 | C | T | 472,174 | 0.00212801 | -0.0147303 | 0.672471 | 4.45E-12 | 9.56E-05 | 45.13549829 |
| **22** | rs78491606 | C | A | 472,174 | 0.00741168 | -0.0756311 | 0.018433 | 1.90E-24 | 0.000206989 | 97.75453964 |
| **23** | rs13062095 | C | T | 472,174 | 0.00214113 | 0.0138552 | 0.327843 | 9.74E-11 | 8.46E-05 | 39.95114305 |
| **24** | rs6776756 | A | G | 472,174 | 0.00203747 | -0.0174439 | 0.597562 | 1.11E-17 | 0.000146352 | 69.11350541 |
| **25** | rs9878436 | T | C | 472,174 | 0.00201819 | -0.0143407 | 0.434393 | 1.20E-12 | 0.000101057 | 47.72131599 |
| **26** | rs112037038 | G | A | 472,174 | 0.00200682 | -0.0167727 | 0.471272 | 6.39E-17 | 0.000140197 | 66.20655989 |
| **27** | rs201296223 | C | T | 472,174 | 0.00376993 | 0.0302737 | 0.078525 | 9.72E-16 | 0.000132633 | 62.63402757 |
| **28** | rs12638862 | G | A | 472,174 | 0.00227875 | -0.0861226 | 0.260773 | 0 | 0.002859596 | 1354.093397 |
| **29** | rs112394943 | C | T | 472,174 | 0.00281641 | -0.0198961 | 0.162741 | 1.61E-12 | 0.000107875 | 50.94127289 |
| **30** | rs2282764 | G | A | 472,174 | 0.00289392 | -0.0224234 | 0.142384 | 9.30E-15 | 0.000122797 | 57.98832324 |
| **31** | rs871134 | T | C | 472,174 | 0.0020263 | -0.0182986 | 0.569032 | 1.71E-19 | 0.000164228 | 77.55664189 |
| **32** | rs10805346 | C | T | 472,174 | 0.00202147 | 0.0117072 | 0.439339 | 6.98E-09 | 6.75E-05 | 31.88348092 |
| **33** | rs4695407 | G | A | 472,174 | 0.00199925 | 0.0141511 | 0.507843 | 1.46E-12 | 0.000100102 | 47.27017807 |
| **34** | rs10024820 | C | T | 472,174 | 0.00205573 | -0.0144438 | 0.389235 | 2.12E-12 | 9.92E-05 | 46.84058016 |
| **35** | rs6536702 | A | G | 472,174 | 0.00238875 | 0.0534148 | 0.774647 | 9.44E-111 | 0.00099614 | 470.8184352 |
| **36** | rs73730598 | A | G | 472,174 | 0.00439272 | 0.0273632 | 0.054793 | 4.69E-10 | 7.76E-05 | 36.62264329 |
| **37** | rs7705526 | A | C | 472,174 | 0.00216124 | 0.0776022 | 0.326578 | 2.43E-282 | 0.002648819 | 1254.019851 |
| **38** | rs61748181 | T | C | 472,174 | 0.00595394 | -0.059181 | 0.028928 | 2.79E-23 | 0.000196773 | 92.92875743 |
| **39** | rs28363070 | A | G | 472,174 | 0.00959987 | 0.0755557 | 0.013389 | 3.53E-15 | 0.00015082 | 71.22366085 |
| **40** | rs6881568 | A | C | 472,174 | 0.00207735 | 0.0169256 | 0.36258 | 3.71E-16 | 0.000132418 | 62.53244565 |
| **41** | rs6873104 | T | A | 472,174 | 0.00335421 | -0.0245329 | 0.101284 | 2.59E-13 | 0.00010957 | 51.74148737 |
| **42** | rs55747751 | A | G | 472,174 | 0.00375164 | -0.0211612 | 0.077375 | 1.70E-08 | 6.39E-05 | 30.19009341 |
| **43** | rs185174247 | A | G | 472,174 | 0.00435145 | 0.0372806 | 0.05609 | 1.06E-17 | 0.000147167 | 69.49858428 |
| **44** | rs80324517 | A | G | 472,174 | 0.00466286 | 0.0396515 | 0.048259 | 1.84E-17 | 0.000144426 | 68.20391316 |
| **45** | rs141127771 | A | G | 472,174 | 0.00268436 | -0.0160204 | 0.259049 | 2.40E-09 | 9.85E-05 | 46.5255093 |
| **46** | rs9398196 | G | A | 472,174 | 0.00201175 | -0.0143586 | 0.52005 | 9.51E-13 | 0.000102919 | 48.60044164 |
| **47** | rs60998424 | G | T | 472,174 | 0.00606553 | -0.0504906 | 0.02903 | 8.49E-17 | 0.000143716 | 67.86823509 |
| **48** | rs13230646 | C | T | 472,174 | 0.00232377 | -0.0173277 | 0.248945 | 8.87E-14 | 0.000112276 | 53.01954289 |
| **49** | rs11769630 | A | T | 472,174 | 0.00389475 | -0.0256807 | 0.072227 | 4.29E-11 | 8.84E-05 | 41.73723107 |
| **50** | rs38664 | C | T | 472,174 | 0.00206433 | -0.0122471 | 0.59665 | 2.98E-09 | 7.22E-05 | 34.09022078 |
| **51** | rs2056726 | A | G | 472,174 | 0.00243638 | -0.0228078 | 0.214376 | 7.87E-21 | 0.000175222 | 82.74924258 |
| **52** | rs7790856 | T | C | 472,174 | 0.00220526 | -0.0437199 | 0.289139 | 1.80E-87 | 0.000785741 | 371.2968676 |
| **53** | rs117630647 | A | G | 472,174 | 0.00720413 | 0.059565 | 0.021346 | 1.36E-16 | 0.000148237 | 70.00395576 |
| **54** | rs117407747 | T | C | 472,174 | 0.00611706 | 0.0450533 | 0.027571 | 1.77E-13 | 0.000108841 | 51.39739777 |
| **55** | rs1985369 | G | A | 472,174 | 0.00300952 | -0.0311893 | 0.868178 | 3.63E-25 | 0.000222658 | 105.1561951 |
| **56** | rs2306646 | C | G | 472,174 | 0.00201898 | -0.0209417 | 0.559475 | 3.31E-25 | 0.000216175 | 102.0937677 |
| **57** | rs762679 | A | T | 472,174 | 0.00285024 | 0.0310104 | 0.856501 | 1.44E-27 | 0.000236386 | 111.6411712 |
| **58** | rs11991877 | A | T | 472,174 | 0.00318686 | -0.030138 | 0.889309 | 3.17E-21 | 0.000178823 | 84.45040754 |
| **59** | rs10112752 | A | G | 472,174 | 0.00202518 | -0.0287522 | 0.430369 | 9.51E-46 | 0.000405328 | 191.4622029 |
| **60** | rs1023767 | A | G | 472,174 | 0.00234772 | -0.0183732 | 0.237595 | 5.04E-15 | 0.000122299 | 57.75319036 |
| **61** | rs10977183 | T | C | 472,174 | 0.00206831 | -0.0136963 | 0.427651 | 3.54E-11 | 9.18E-05 | 43.36377168 |
| **62** | rs11557154 | T | C | 472,174 | 0.00298538 | -0.0343719 | 0.13003 | 1.13E-30 | 0.000267291 | 126.2412373 |
| **63** | rs4743037 | T | C | 472,174 | 0.00238094 | 0.0147971 | 0.230874 | 5.14E-10 | 7.78E-05 | 36.71890614 |
| **64** | rs10905255 | T | G | 472,174 | 0.00203099 | -0.0182493 | 0.57919 | 2.58E-19 | 0.000162341 | 76.66555128 |
| **65** | rs7099229 | A | G | 472,174 | 0.00224403 | -0.0153288 | 0.273301 | 8.44E-12 | 9.33E-05 | 44.07403995 |
| **66** | rs117034449 | A | G | 472,174 | 0.00667898 | 0.0374377 | 0.023304 | 2.08E-08 | 6.38E-05 | 30.12771093 |
| **67** | rs6584579 | G | A | 472,174 | 0.00204674 | 0.0114923 | 0.398876 | 1.97E-08 | 6.33E-05 | 29.90705263 |
| **68** | rs9419958 | C | T | 472,174 | 0.00293847 | -0.0810098 | 0.86139 | 2.64E-167 | 0.00156711 | 741.106898 |
| **69** | rs77231040 | C | G | 472,174 | 0.0134649 | 0.0989303 | 0.005742 | 2.02E-13 | 0.000111751 | 52.77153114 |
| **70** | rs939916 | A | G | 472,174 | 0.00216724 | 0.0241795 | 0.669967 | 6.63E-29 | 0.000258545 | 122.1090732 |
| **71** | rs10768683 | G | C | 472,174 | 0.00277015 | 0.0469922 | 0.841033 | 1.52E-64 | 0.000590475 | 278.9705196 |
| **72** | rs10840270 | G | C | 472,174 | 0.00212494 | 0.014383 | 0.655684 | 1.30E-11 | 9.34E-05 | 44.10842443 |
| **73** | rs2293579 | A | G | 472,174 | 0.00205481 | -0.012915 | 0.386274 | 3.27E-10 | 7.91E-05 | 37.34421997 |
| **74** | rs611646 | A | T | 472,174 | 0.00203547 | -0.0368309 | 0.408685 | 3.52E-73 | 0.000655635 | 309.7756766 |
| **75** | rs11212631 | C | T | 472,174 | 0.00256554 | -0.0193458 | 0.199229 | 4.68E-14 | 0.000119416 | 56.39185352 |
| **76** | rs10845387 | A | G | 472,174 | 0.00209396 | -0.0141214 | 0.352666 | 1.54E-11 | 9.10E-05 | 42.99493428 |
| **77** | rs12369950 | C | T | 472,174 | 0.00290205 | -0.0178308 | 0.140675 | 8.04E-10 | 7.69E-05 | 36.29774439 |
| **78** | rs79755767 | A | G | 472,174 | 0.00343286 | 0.0278665 | 0.095708 | 4.76E-16 | 0.000134416 | 63.47612193 |
| **79** | rs17445108 | A | G | 472,174 | 0.00300983 | -0.0168922 | 0.12695 | 2.00E-08 | 6.33E-05 | 29.86771149 |
| **80** | rs1907702 | A | G | 472,174 | 0.00242651 | 0.0150247 | 0.766771 | 5.94E-10 | 8.07E-05 | 38.12634201 |
| **81** | rs76666449 | C | T | 472,174 | 0.00333186 | 0.0295125 | 0.100625 | 8.17E-19 | 0.000157648 | 74.44874845 |
| **82** | rs10773176 | G | A | 472,174 | 0.00228534 | -0.0172009 | 0.741214 | 5.21E-14 | 0.000113505 | 53.60018103 |
| **83** | rs28577594 | C | G | 472,174 | 0.00224024 | 0.0187657 | 0.709829 | 5.45E-17 | 0.000145067 | 68.50630041 |
| **84** | rs1332941 | G | A | 472,174 | 0.00273159 | 0.0256552 | 0.820466 | 5.88E-21 | 0.000193905 | 91.57407703 |
| **85** | rs670180 | A | T | 472,174 | 0.00203062 | -0.0115801 | 0.569104 | 1.18E-08 | 6.58E-05 | 31.0561424 |
| **86** | rs9600019 | T | C | 472,174 | 0.00213096 | 0.0127134 | 0.335579 | 2.43E-09 | 7.21E-05 | 34.03479017 |
| **87** | rs3093888 | A | G | 472,174 | 0.00452459 | -0.028973 | 0.05131 | 1.52E-10 | 8.17E-05 | 38.59037411 |
| **88** | rs73581419 | T | C | 472,174 | 0.00324156 | 0.0229838 | 0.106605 | 1.34E-12 | 0.000100622 | 47.51587018 |
| **89** | rs113525195 | A | C | 472,174 | 0.00224132 | -0.0124075 | 0.290254 | 3.10E-08 | 6.34E-05 | 29.95073657 |
| **90** | rs762810 | A | C | 472,174 | 0.00210014 | -0.0202965 | 0.352486 | 4.27E-22 | 0.000188046 | 88.80659967 |
| **91** | rs137901416 | A | G | 472,174 | 0.00332355 | 0.04572 | 0.100311 | 4.66E-43 | 0.000377297 | 178.2163432 |
| **92** | rs8006485 | T | G | 472,174 | 0.00200238 | 0.0191453 | 0.453102 | 1.16E-21 | 0.000181659 | 85.78982704 |
| **93** | rs1957937 | T | A | 472,174 | 0.00273361 | 0.0209365 | 0.16018 | 1.88E-14 | 0.000117932 | 55.69088391 |
| **94** | rs17677991 | G | C | 472,174 | 0.00210806 | 0.0222664 | 0.342123 | 4.45E-26 | 0.000223181 | 105.403286 |
| **95** | rs113119217 | A | T | 472,174 | 0.00234274 | -0.0332464 | 0.240708 | 1.04E-45 | 0.000404035 | 190.8509521 |
| **96** | rs7164950 | G | A | 472,174 | 0.00204001 | 0.0129362 | 0.405979 | 2.28E-10 | 8.07E-05 | 38.11396152 |
| **97** | rs5742915 | C | T | 472,174 | 0.00202886 | 0.0193377 | 0.445829 | 1.55E-21 | 0.000184779 | 87.26341964 |
| **98** | rs112511042 | C | T | 472,174 | 0.00416402 | -0.0351434 | 0.062005 | 3.18E-17 | 0.000143663 | 67.84336933 |
| **99** | rs12932179 | G | A | 472,174 | 0.0020276 | -0.0136257 | 0.561399 | 1.82E-11 | 9.14E-05 | 43.17465068 |
| **100** | rs182059586 | C | T | 472,174 | 0.00680853 | -0.0571159 | 0.02511 | 4.91E-17 | 0.000159715 | 75.42511339 |
| **101** | rs450962 | G | A | 472,174 | 0.00245428 | 0.0142833 | 0.283779 | 5.89E-09 | 8.29E-05 | 39.16071961 |
| **102** | rs1105407 | G | C | 472,174 | 0.0027778 | 0.0162208 | 0.167323 | 5.24E-09 | 7.33E-05 | 34.62093705 |
| **103** | rs76219171 | A | G | 472,174 | 0.00431741 | 0.0359839 | 0.058433 | 7.78E-17 | 0.000142481 | 67.28495767 |
| **104** | rs3785074 | G | A | 472,174 | 0.00220455 | 0.023863 | 0.289672 | 2.64E-27 | 0.00023434 | 110.6744938 |
| **105** | rs62050964 | A | G | 472,174 | 0.00220429 | -0.0193559 | 0.337645 | 1.62E-18 | 0.000167574 | 79.13723814 |
| **106** | rs11866592 | A | G | 472,174 | 0.00286611 | 0.03464 | 0.14198 | 1.25E-33 | 0.000292355 | 138.0821567 |
| **107** | rs2967355 | C | A | 472,174 | 0.00238972 | -0.0461595 | 0.774276 | 3.95E-83 | 0.000744776 | 351.9245186 |
| **108** | rs11117354 | C | T | 472,174 | 0.00219601 | 0.0232506 | 0.696513 | 3.40E-26 | 0.000228543 | 107.936204 |
| **109** | rs12925933 | C | A | 472,174 | 0.00213796 | -0.0146622 | 0.66221 | 6.98E-12 | 9.62E-05 | 45.4164096 |
| **110** | rs59409453 | G | A | 472,174 | 0.00230175 | 0.0202133 | 0.730602 | 1.61E-18 | 0.000160835 | 75.9538422 |
| **111** | rs12451892 | C | T | 472,174 | 0.00207578 | -0.0116145 | 0.380511 | 2.20E-08 | 6.36E-05 | 30.03030186 |
| **112** | rs4724 | A | G | 472,174 | 0.00312441 | -0.0547446 | 0.116598 | 9.81E-69 | 0.000617393 | 291.6960121 |
| **113** | rs111527438 | C | T | 472,174 | 0.00211016 | 0.0125 | 0.351251 | 3.15E-09 | 7.12E-05 | 33.62601865 |
| **114** | rs56799554 | G | A | 472,174 | 0.00267858 | -0.0259793 | 0.170183 | 3.05E-22 | 0.000190627 | 90.02568079 |
| **115** | rs7209057 | A | G | 472,174 | 0.00202865 | 0.011819 | 0.561044 | 5.68E-09 | 6.88E-05 | 32.48923493 |
| **116** | rs144204502 | T | C | 472,174 | 0.00913369 | -0.100574 | 0.012562 | 3.37E-28 | 0.00025094 | 118.5166296 |
| **117** | rs7221585 | T | C | 472,174 | 0.00247042 | 0.0143271 | 0.22401 | 6.65E-09 | 7.14E-05 | 33.69778335 |
| **118** | rs3891167 | G | A | 472,174 | 0.00239551 | -0.0425685 | 0.253435 | 1.20E-70 | 0.000685711 | 323.9955457 |
| **119** | rs150150565 | T | C | 472,174 | 0.00739877 | 0.063762 | 0.021455 | 6.82E-18 | 0.000170712 | 80.61902727 |
| **120** | rs116863223 | A | G | 472,174 | 0.00937157 | -0.0817874 | 0.011763 | 2.61E-18 | 0.000155518 | 73.44289421 |
| **121** | rs139669835 | T | C | 472,174 | 0.0105346 | -0.0612563 | 0.009365 | 6.07E-09 | 6.96E-05 | 32.87633786 |
| **122** | rs28481848 | G | A | 472,174 | 0.00285068 | -0.0299462 | 0.143727 | 8.20E-26 | 0.000220731 | 104.2461802 |
| **123** | rs2276182 | G | C | 472,174 | 0.00204247 | 0.0233529 | 0.403227 | 2.84E-30 | 0.000262464 | 123.9608761 |
| **124** | rs9955360 | A | C | 472,174 | 0.00299791 | -0.0190311 | 0.869288 | 2.18E-10 | 8.23E-05 | 38.8662732 |
| **125** | rs11085072 | T | C | 472,174 | 0.00236713 | -0.0131806 | 0.236909 | 2.57E-08 | 6.28E-05 | 29.66099789 |
| **126** | rs8105767 | G | A | 472,174 | 0.00220117 | 0.0328384 | 0.29467 | 2.49E-50 | 0.000448252 | 211.7469673 |
| **127** | rs4530278 | T | G | 472,174 | 0.0020567 | 0.0138793 | 0.59815 | 1.50E-11 | 9.26E-05 | 43.73001753 |
| **128** | rs429358 | C | T | 472,174 | 0.00277091 | 0.0173498 | 0.153969 | 3.82E-10 | 7.84E-05 | 37.03162725 |
| **129** | rs8102497 | A | G | 472,174 | 0.0020233 | -0.0149654 | 0.431828 | 1.40E-13 | 0.0001099 | 51.89735711 |
| **130** | rs6054257 | A | G | 472,174 | 0.00247729 | -0.0141684 | 0.793522 | 1.07E-08 | 6.58E-05 | 31.06226855 |
| **131** | rs11699829 | A | G | 472,174 | 0.00602028 | 0.0641957 | 0.034146 | 1.51E-26 | 0.000271827 | 128.3841739 |
| **132** | rs143190905 | T | G | 472,174 | 0.00369421 | -0.0723995 | 0.080404 | 1.61E-85 | 0.000775132 | 366.2797034 |
| **133** | rs35640778 | A | G | 472,174 | 0.00702087 | -0.209011 | 0.020757 | 9.57E-195 | 0.00177592 | 840.03142 |
| **134** | rs41304832 | A | G | 472,174 | 0.0093095 | 0.0611702 | 0.012378 | 5.01E-11 | 9.15E-05 | 43.20072085 |
| **135** | rs142426306 | T | C | 472,174 | 0.00539933 | -0.0504903 | 0.039544 | 8.66E-21 | 0.000193644 | 91.45096777 |
| **136** | rs117512405 | A | G | 472,174 | 0.00824611 | -0.0790134 | 0.017043 | 9.53E-22 | 0.000209176 | 98.78776115 |
| **137** | rs28502153 | A | C | 472,174 | 0.00206208 | -0.0215916 | 0.377958 | 1.18E-25 | 0.000219211 | 103.5281233 |
| **138** | rs6007020 | C | T | 472,174 | 0.00209637 | 0.0144904 | 0.367823 | 4.77E-12 | 9.76E-05 | 46.1116813 |
| **139** | rs131794 | C | A | 472,174 | 0.00246292 | -0.0247859 | 0.792653 | 8.00E-24 | 0.000201939 | 95.36921005 |
| **140** | rs1003322 | A | C | 472,174 | 0.00247546 | 0.0141734 | 0.213742 | 1.03E-08 | 6.75E-05 | 31.88324995 |

SNP, Single Nucleotide Polymorphism; EA, Effect Allele; OA, Other Allele; EAF, Effect Allele Frequency; SE, Standard Error; TL, Telomere Length.

**Supplementary Table S2** Information of identified SNPs in exposure (TL) and outcomes (AIT).

|  |  | | | **Exposure (TL)** | | | **Outcome (AIT)** | | | | |
| --- | --- | --- | --- | --- | --- | --- | --- | --- | --- | --- | --- |
|  | **SNP** | **EA** | **OA** | **β** | **SE** | **p-value** | **Case** | **Control** | **β** | **SE** | **p-value** |
| **1** | rs10024820 | C | T | -0.0144438 | 0.00205573 | 2.12E-12 | 539 | 349,717 | 0.142549 | 0.0617936 | 0.0210625 |
| **2** | rs1003322 | A | C | 0.0141734 | 0.00247546 | 1.03E-08 | 539 | 349,717 | 0.0160292 | 0.0871728 | 0.854108 |
| **3** | rs10112752 | A | G | -0.0287522 | 0.00202518 | 9.51E-46 | 539 | 349,717 | 0.00964463 | 0.0616884 | 0.875762 |
| **4** | rs10768683 | G | C | 0.0469922 | 0.00277015 | 1.52E-64 | 539 | 349,717 | 0.054995 | 0.0758694 | 0.468536 |
| **5** | rs10773176 | G | A | -0.0172009 | 0.00228534 | 5.21E-14 | 539 | 349,717 | 0.140804 | 0.0779353 | 0.0708125 |
| **6** | rs10805346 | C | T | 0.0117072 | 0.00202147 | 6.98E-09 | 539 | 349,717 | -0.00361444 | 0.0617439 | 0.953319 |
| **7** | rs10840270 | G | C | 0.014383 | 0.00212494 | 1.30E-11 | 539 | 349,717 | -0.0618372 | 0.0652942 | 0.343611 |
| **8** | rs10845387 | A | G | -0.0141214 | 0.00209396 | 1.54E-11 | 539 | 349,717 | 0.0769359 | 0.0658342 | 0.242552 |
| **9** | rs10905255 | T | G | -0.0182493 | 0.00203099 | 2.58E-19 | 539 | 349,717 | 0.161102 | 0.0609726 | 0.00823683 |
| **10** | rs10977183 | T | C | -0.0136963 | 0.00206831 | 3.54E-11 | 539 | 349,717 | -0.0495808 | 0.0625459 | 0.427946 |
| **11** | rs1105407 | G | C | 0.0162208 | 0.0027778 | 5.24E-09 | 539 | 349,717 | -0.117766 | 0.0806965 | 0.144464 |
| **12** | rs11085072 | T | C | -0.0131806 | 0.00236713 | 2.57E-08 | 539 | 349,717 | -0.0432274 | 0.0826495 | 0.600961 |
| **13** | rs11117354 | C | T | 0.0232506 | 0.00219601 | 3.40E-26 | 539 | 349,717 | -0.0341279 | 0.0619918 | 0.581961 |
| **14** | rs111527438 | C | T | 0.0125 | 0.00211016 | 3.15E-09 | 539 | 349,717 | 0.0516485 | 0.0654628 | 0.430126 |
| **15** | rs112037038 | G | A | -0.0167727 | 0.00200682 | 6.39E-17 | 539 | 349,717 | -0.0259329 | 0.0612982 | 0.67225 |
| **16** | rs112394943 | C | T | -0.0198961 | 0.00281641 | 1.61E-12 | 539 | 349,717 | -0.0241348 | 0.073339 | 0.742091 |
| **17** | rs112511042 | C | T | -0.0351434 | 0.00416402 | 3.18E-17 | 539 | 349,717 | -0.147086 | 0.114275 | 0.198051 |
| **18** | rs113119217 | A | T | -0.0332464 | 0.00234274 | 1.04E-45 | 539 | 349,717 | 0.0703021 | 0.0720766 | 0.329371 |
| **19** | rs113525195 | A | C | -0.0124075 | 0.00224132 | 3.10E-08 | 539 | 349,717 | -0.0394332 | 0.066744 | 0.554646 |
| **20** | rs11557154 | T | C | -0.0343719 | 0.00298538 | 1.13E-30 | 539 | 349,717 | -0.186777 | 0.0907844 | 0.0396515 |
| **21** | rs11579626 | C | A | 0.0265113 | 0.00357752 | 1.26E-13 | 539 | 349,717 | -0.0238449 | 0.0948151 | 0.801437 |
| **22** | rs11584821 | T | C | -0.0306517 | 0.00263623 | 3.00E-31 | 539 | 349,717 | -0.0936345 | 0.0795921 | 0.239423 |
| **23** | rs116863223 | A | G | -0.0817874 | 0.00937157 | 2.61E-18 | 539 | 349,717 | -0.140627 | 0.242384 | 0.56179 |
| **24** | rs11699829 | A | G | 0.0641957 | 0.00602028 | 1.51E-26 | 539 | 349,717 | -0.152028 | 0.25134 | 0.545265 |
| **25** | rs117034449 | A | G | 0.0374377 | 0.00667898 | 2.08E-08 | 539 | 349,717 | -0.147404 | 0.249141 | 0.554086 |
| **26** | rs117407747 | T | C | 0.0450533 | 0.00611706 | 1.77E-13 | 539 | 349,717 | -0.487849 | 0.29552 | 0.0987757 |
| **27** | rs117512405 | A | G | -0.0790134 | 0.00824611 | 9.53E-22 | 539 | 349,717 | -0.050799 | 0.136193 | 0.709153 |
| **28** | rs117630647 | A | G | 0.059565 | 0.00720413 | 1.36E-16 | 539 | 349,717 | -0.0297643 | 0.242176 | 0.902183 |
| **29** | rs11769630 | A | T | -0.0256807 | 0.00389475 | 4.29E-11 | 539 | 349,717 | -0.107607 | 0.101902 | 0.290972 |
| **30** | rs11866592 | A | G | 0.03464 | 0.00286611 | 1.25E-33 | 539 | 349,717 | 0.0999876 | 0.078928 | 0.205219 |
| **31** | rs11991877 | A | T | -0.030138 | 0.00318686 | 3.17E-21 | 539 | 349,717 | 0.0573297 | 0.0882783 | 0.516067 |
| **32** | rs12369950 | C | T | -0.0178308 | 0.00290205 | 8.04E-10 | 539 | 349,717 | 0.0505903 | 0.0863052 | 0.557754 |
| **33** | rs12412214 | A | G | -0.0245174 | 0.00222685 | 3.42E-28 | 539 | 349,717 | 0.0413285 | 0.0625464 | 0.508762 |
| **34** | rs12451892 | C | T | -0.0116145 | 0.00207578 | 2.20E-08 | 539 | 349,717 | 0.0333424 | 0.0623033 | 0.592538 |
| **35** | rs12619538 | C | A | 0.0167489 | 0.00287813 | 5.91E-09 | 539 | 349,717 | 0.146658 | 0.0882713 | 0.0966229 |
| **36** | rs12925933 | C | A | -0.0146622 | 0.00213796 | 6.98E-12 | 539 | 349,717 | 0.0591678 | 0.0617688 | 0.338118 |
| **37** | rs12932179 | G | A | -0.0136257 | 0.0020276 | 1.82E-11 | 539 | 349,717 | 0.0458211 | 0.0627454 | 0.465225 |
| **38** | rs13062095 | C | T | 0.0138552 | 0.00214113 | 9.74E-11 | 539 | 349,717 | 0.0346949 | 0.0633545 | 0.583945 |
| **39** | rs131794 | C | A | -0.0247859 | 0.00246292 | 8.00E-24 | 539 | 349,717 | -0.111167 | 0.0790903 | 0.159851 |
| **40** | rs13230646 | C | T | -0.0173277 | 0.00232377 | 8.87E-14 | 539 | 349,717 | 0.15917 | 0.0803279 | 0.0475346 |
| **41** | rs1332941 | G | A | 0.0256552 | 0.00273159 | 5.88E-21 | 539 | 349,717 | 0.0868645 | 0.0762197 | 0.254428 |
| **42** | rs137901416 | A | G | 0.04572 | 0.00332355 | 4.66E-43 | 539 | 349,717 | -0.0292381 | 0.122796 | 0.8118 |
| **43** | rs139669835 | T | C | -0.0612563 | 0.0105346 | 6.07E-09 | 539 | 349,717 | 0.453662 | 0.865746 | 0.600269 |
| **44** | rs139795227 | C | A | 0.0599379 | 0.00873247 | 6.71E-12 | 539 | 349,717 | -0.124162 | 0.192399 | 0.51871 |
| **45** | rs142426306 | T | C | -0.0504903 | 0.00539933 | 8.66E-21 | 539 | 349,717 | -0.23972 | 0.154116 | 0.11984 |
| **46** | rs143190905 | T | G | -0.0723995 | 0.00369421 | 1.61E-85 | 539 | 349,717 | 0.0109078 | 0.116964 | 0.925699 |
| **47** | rs144204502 | T | C | -0.100574 | 0.00913369 | 3.37E-28 | 539 | 349,717 | -0.279346 | 0.162916 | 0.0864072 |
| **48** | rs150150565 | T | C | 0.063762 | 0.00739877 | 6.82E-18 | 539 | 349,717 | 0.24531 | 0.222412 | 0.270048 |
| **49** | rs17445108 | A | G | -0.0168922 | 0.00300983 | 2.00E-08 | 539 | 349,717 | -0.114412 | 0.0925376 | 0.216315 |
| **50** | rs17677991 | G | C | 0.0222664 | 0.00210806 | 4.45E-26 | 539 | 349,717 | 0.106431 | 0.0626519 | 0.0893635 |
| **51** | rs182059586 | C | T | -0.0571159 | 0.00680853 | 4.91E-17 | 539 | 349,717 | 0.776052 | 0.357147 | 0.0297865 |
| **52** | rs185174247 | A | G | 0.0372806 | 0.00435145 | 1.06E-17 | 539 | 349,717 | -0.139142 | 0.173088 | 0.421467 |
| **53** | rs188918174 | T | C | 0.0403062 | 0.00543604 | 1.22E-13 | 539 | 349,717 | -0.108538 | 0.146014 | 0.457276 |
| **54** | rs1907702 | A | G | 0.0150247 | 0.00242651 | 5.94E-10 | 539 | 349,717 | -0.0721886 | 0.0765569 | 0.345712 |
| **55** | rs1957937 | T | A | 0.0209365 | 0.00273361 | 1.88E-14 | 539 | 349,717 | 0.165235 | 0.07926 | 0.0370937 |
| **56** | rs1985369 | G | A | -0.0311893 | 0.00300952 | 3.63E-25 | 539 | 349,717 | -0.047733 | 0.0988464 | 0.629166 |
| **57** | rs2056726 | A | G | -0.0228078 | 0.00243638 | 7.87E-21 | 539 | 349,717 | 0.00703567 | 0.0732286 | 0.923459 |
| **58** | rs2282764 | G | A | -0.0224234 | 0.00289392 | 9.30E-15 | 539 | 349,717 | -0.0920484 | 0.0875983 | 0.29335 |
| **59** | rs2293579 | A | G | -0.012915 | 0.00205481 | 3.27E-10 | 539 | 349,717 | 0.0686925 | 0.0664794 | 0.301468 |
| **60** | rs2555104 | C | A | -0.0139717 | 0.00203498 | 6.61E-12 | 539 | 349,717 | -0.0565778 | 0.0612996 | 0.356022 |
| **61** | rs28363070 | A | G | 0.0755557 | 0.00959987 | 3.53E-15 | 539 | 349,717 | -0.613319 | 0.695856 | 0.378108 |
| **62** | rs28481848 | G | A | -0.0299462 | 0.00285068 | 8.20E-26 | 539 | 349,717 | 0.00661731 | 0.11219 | 0.952966 |
| **63** | rs28502153 | A | C | -0.0215916 | 0.00206208 | 1.18E-25 | 539 | 349,717 | -0.0223211 | 0.0631236 | 0.723632 |
| **64** | rs28577594 | C | G | 0.0187657 | 0.00224024 | 5.45E-17 | 539 | 349,717 | 0.0580413 | 0.0675699 | 0.390351 |
| **65** | rs2967355 | C | A | -0.0461595 | 0.00238972 | 3.95E-83 | 539 | 349,717 | -0.00126256 | 0.0803476 | 0.987463 |
| **66** | rs2977608 | C | A | 0.0129483 | 0.00233716 | 3.02E-08 | 539 | 349,717 | 0.084501 | 0.0665271 | 0.204022 |
| **67** | rs3093888 | A | G | -0.028973 | 0.00452459 | 1.52E-10 | 539 | 349,717 | -0.0915216 | 0.108785 | 0.400178 |
| **68** | rs35640778 | A | G | -0.209011 | 0.00702087 | 9.57E-195 | 539 | 349,717 | 0.416368 | 0.268575 | 0.121073 |
| **69** | rs3767952 | A | G | 0.0134472 | 0.00238826 | 1.80E-08 | 539 | 349,717 | -0.0153058 | 0.0735279 | 0.835102 |
| **70** | rs3785074 | G | A | 0.023863 | 0.00220455 | 2.64E-27 | 539 | 349,717 | 0.0301202 | 0.0754221 | 0.689631 |
| **71** | rs38664 | C | T | -0.0122471 | 0.00206433 | 2.98E-09 | 539 | 349,717 | -0.0393908 | 0.0616333 | 0.522749 |
| **72** | rs3891167 | G | A | -0.0425685 | 0.00239551 | 1.20E-70 | 539 | 349,717 | -0.0553667 | 0.0705613 | 0.432652 |
| **73** | rs41269079 | A | T | 0.0153617 | 0.0025499 | 1.70E-09 | 539 | 349,717 | 0.126019 | 0.0752275 | 0.093901 |
| **74** | rs41304832 | A | G | 0.0611702 | 0.0093095 | 5.01E-11 | 539 | 349,717 | 0.0425313 | 0.16306 | 0.794222 |
| **75** | rs429358 | C | T | 0.0173498 | 0.00277091 | 3.82E-10 | 539 | 349,717 | 0.017597 | 0.0797581 | 0.82538 |
| **76** | rs4498805 | T | G | 0.0150601 | 0.00200376 | 5.65E-14 | 539 | 349,717 | -0.0502122 | 0.0611094 | 0.411261 |
| **77** | rs4530278 | T | G | 0.0138793 | 0.0020567 | 1.50E-11 | 539 | 349,717 | 0.101429 | 0.062548 | 0.104885 |
| **78** | rs4695407 | G | A | 0.0141511 | 0.00199925 | 1.46E-12 | 539 | 349,717 | 0.0289277 | 0.0613069 | 0.637033 |
| **79** | rs4724 | A | G | -0.0547446 | 0.00312441 | 9.81E-69 | 539 | 349,717 | -0.0351254 | 0.099153 | 0.723148 |
| **80** | rs4743037 | T | C | 0.0147971 | 0.00238094 | 5.14E-10 | 539 | 349,717 | -0.0200567 | 0.0744174 | 0.787532 |
| **81** | rs55747751 | A | G | -0.0211612 | 0.00375164 | 1.70E-08 | 539 | 349,717 | -0.0518083 | 0.131633 | 0.693891 |
| **82** | rs56799554 | G | A | -0.0259793 | 0.00267858 | 3.05E-22 | 539 | 349,717 | 0.0279158 | 0.0726007 | 0.7006 |
| **83** | rs5742915 | C | T | 0.0193377 | 0.00202886 | 1.55E-21 | 539 | 349,717 | -0.0873616 | 0.0624255 | 0.161677 |
| **84** | rs59409453 | G | A | 0.0202133 | 0.00230175 | 1.61E-18 | 539 | 349,717 | -0.00634745 | 0.0753329 | 0.932851 |
| **85** | rs6007020 | C | T | 0.0144904 | 0.00209637 | 4.77E-12 | 539 | 349,717 | -0.0484012 | 0.0638877 | 0.448692 |
| **86** | rs6054257 | A | G | -0.0141684 | 0.00247729 | 1.07E-08 | 539 | 349,717 | 0.0386642 | 0.0767767 | 0.614547 |
| **87** | rs611646 | A | T | -0.0368309 | 0.00203547 | 3.52E-73 | 539 | 349,717 | -0.0321882 | 0.062458 | 0.606303 |
| **88** | rs61748181 | T | C | -0.059181 | 0.00595394 | 2.79E-23 | 539 | 349,717 | 0.0992157 | 0.137737 | 0.471323 |
| **89** | rs6536702 | A | G | 0.0534148 | 0.00238875 | 9.44E-111 | 539 | 349,717 | -0.0816885 | 0.078422 | 0.297573 |
| **90** | rs6584579 | G | A | 0.0114923 | 0.00204674 | 1.97E-08 | 539 | 349,717 | -0.0195678 | 0.0652884 | 0.764395 |
| **91** | rs6587577 | G | A | -0.0182148 | 0.0026359 | 4.84E-12 | 539 | 349,717 | 0.141708 | 0.0836202 | 0.0901384 |
| **92** | rs6659669 | T | C | -0.0117091 | 0.00205167 | 1.15E-08 | 539 | 349,717 | 0.0138805 | 0.0619419 | 0.822688 |
| **93** | rs6669563 | A | G | 0.0182358 | 0.00202476 | 2.13E-19 | 539 | 349,717 | -0.108647 | 0.0615 | 0.077293 |
| **94** | rs66731853 | A | G | -0.0177791 | 0.00215421 | 1.54E-16 | 539 | 349,717 | 0.0815451 | 0.0768724 | 0.288787 |
| **95** | rs6751209 | C | T | -0.0140465 | 0.00248465 | 1.57E-08 | 539 | 349,717 | -0.00441887 | 0.0794705 | 0.955657 |
| **96** | rs6776756 | A | G | -0.0174439 | 0.00203747 | 1.11E-17 | 539 | 349,717 | 0.0815323 | 0.0619417 | 0.188082 |
| **97** | rs6873104 | T | A | -0.0245329 | 0.00335421 | 2.59E-13 | 539 | 349,717 | -0.196828 | 0.0999311 | 0.048881 |
| **98** | rs6881568 | A | C | 0.0169256 | 0.00207735 | 3.71E-16 | 539 | 349,717 | -0.0672808 | 0.0684185 | 0.325425 |
| **99** | rs7099229 | A | G | -0.0153288 | 0.00224403 | 8.44E-12 | 539 | 349,717 | -0.0133652 | 0.079946 | 0.86723 |
| **100** | rs7164950 | G | A | 0.0129362 | 0.00204001 | 2.28E-10 | 539 | 349,717 | -0.0522261 | 0.0622644 | 0.401594 |
| **101** | rs7209057 | A | G | 0.011819 | 0.00202865 | 5.68E-09 | 539 | 349,717 | -0.0462325 | 0.0616407 | 0.453236 |
| **102** | rs7221585 | T | C | 0.0143271 | 0.00247042 | 6.65E-09 | 539 | 349,717 | 0.100986 | 0.067969 | 0.137343 |
| **103** | rs73581419 | T | C | 0.0229838 | 0.00324156 | 1.34E-12 | 539 | 349,717 | -0.130526 | 0.11068 | 0.238276 |
| **104** | rs73730598 | A | G | 0.0273632 | 0.00439272 | 4.69E-10 | 539 | 349,717 | 0.151404 | 0.123049 | 0.218534 |
| **105** | rs7555872 | G | A | 0.027267 | 0.00498734 | 4.57E-08 | 539 | 349,717 | -0.100552 | 0.156386 | 0.520242 |
| **106** | rs76219171 | A | G | 0.0359839 | 0.00431741 | 7.78E-17 | 539 | 349,717 | 0.175989 | 0.166642 | 0.290926 |
| **107** | rs762679 | A | T | 0.0310104 | 0.00285024 | 1.44E-27 | 539 | 349,717 | 0.00464569 | 0.0814558 | 0.954519 |
| **108** | rs762810 | A | C | -0.0202965 | 0.00210014 | 4.27E-22 | 539 | 349,717 | 0.0271193 | 0.0624794 | 0.664251 |
| **109** | rs76666449 | C | T | 0.0295125 | 0.00333186 | 8.17E-19 | 539 | 349,717 | 0.0695699 | 0.0895565 | 0.43726 |
| **110** | rs77231040 | C | G | 0.0989303 | 0.0134649 | 2.02E-13 | 539 | 349,717 | 0.178338 | 0.189339 | 0.346245 |
| **111** | rs7772289 | T | G | 0.017549 | 0.00200003 | 1.72E-18 | 539 | 349,717 | -0.012307 | 0.062751 | 0.844513 |
| **112** | rs7790856 | T | C | -0.0437199 | 0.00220526 | 1.80E-87 | 539 | 349,717 | -0.0690162 | 0.0732085 | 0.345817 |
| **113** | rs78491606 | C | A | -0.0756311 | 0.00741168 | 1.90E-24 | 539 | 349,717 | -0.000737189 | 0.186679 | 0.996849 |
| **114** | rs79755767 | A | G | 0.0278665 | 0.00343286 | 4.76E-16 | 539 | 349,717 | -0.0508476 | 0.103432 | 0.622997 |
| **115** | rs8006485 | T | G | 0.0191453 | 0.00200238 | 1.16E-21 | 539 | 349,717 | 0.0439982 | 0.0630397 | 0.485211 |
| **116** | rs80324517 | A | G | 0.0396515 | 0.00466286 | 1.84E-17 | 539 | 349,717 | -0.18443 | 0.125723 | 0.142388 |
| **117** | rs8102497 | A | G | -0.0149654 | 0.0020233 | 1.40E-13 | 539 | 349,717 | -0.0552985 | 0.0615799 | 0.369188 |
| **118** | rs8105767 | G | A | 0.0328384 | 0.00220117 | 2.49E-50 | 539 | 349,717 | 0.0192723 | 0.0659062 | 0.769965 |
| **119** | rs869785 | C | T | -0.0147303 | 0.00212801 | 4.45E-12 | 539 | 349,717 | -0.0614162 | 0.0647337 | 0.342746 |
| **120** | rs871134 | T | C | -0.0182986 | 0.0020263 | 1.71E-19 | 539 | 349,717 | -0.108431 | 0.0615156 | 0.0779579 |
| **121** | rs932002 | T | C | -0.0402052 | 0.00279667 | 7.31E-47 | 539 | 349,717 | -0.017073 | 0.0718912 | 0.812281 |
| **122** | rs9398196 | G | A | -0.0143586 | 0.00201175 | 9.51E-13 | 539 | 349,717 | -0.0795874 | 0.0629702 | 0.20627 |
| **123** | rs939916 | A | G | 0.0241795 | 0.00216724 | 6.63E-29 | 539 | 349,717 | 0.0989077 | 0.0695754 | 0.155145 |
| **124** | rs9419958 | C | T | -0.0810098 | 0.00293847 | 2.64E-167 | 539 | 349,717 | -0.11514 | 0.0952584 | 0.226774 |
| **125** | rs9600019 | T | C | 0.0127134 | 0.00213096 | 2.43E-09 | 539 | 349,717 | 0.00112938 | 0.0633652 | 0.98578 |
| **126** | rs965109 | T | C | -0.101702 | 0.00648201 | 1.77E-55 | 539 | 349,717 | -0.0916456 | 0.118123 | 0.43784 |
| **127** | rs9878436 | T | C | -0.0143407 | 0.00201819 | 1.20E-12 | 539 | 349,717 | -0.0479183 | 0.0639864 | 0.453928 |
| **128** | rs9955360 | A | C | -0.0190311 | 0.00299791 | 2.18E-10 | 539 | 349,717 | -0.09605 | 0.0871564 | 0.270444 |

SNP, Single Nucleotide Polymorphism; EA, Effect Allele; OA, Other Allele; SE, Standard Error; TL, Telomere Length; AIT, Autoimmune Thyroiditis.

**Supplementary Table S3** Information of identified SNPs in exposure (TL) and outcomes (GD).

|  |  | | | **Exposure (TL)** | | | **Outcome (GD)** | | | | |
| --- | --- | --- | --- | --- | --- | --- | --- | --- | --- | --- | --- |
|  | **SNP** | **EA** | **OA** | **β** | **SE** | **p-value** | **Case** | **Control** | **β** | **SE** | **p-value** |
| **1** | rs10024820 | C | T | -0.0144438 | 0.00205573 | 2.12E-12 | 3,176 | 409,005 | -0.0213179 | 0.0256779 | 0.406424 |
| **2** | rs1003322 | A | C | 0.0141734 | 0.00247546 | 1.03E-08 | 3,176 | 409,005 | -0.0360578 | 0.036156 | 0.318626 |
| **3** | rs10112752 | A | G | -0.0287522 | 0.00202518 | 9.51E-46 | 3,176 | 409,005 | 0.00536922 | 0.0255808 | 0.833751 |
| **4** | rs10768683 | G | C | 0.0469922 | 0.00277015 | 1.52E-64 | 3,176 | 409,005 | 0.01259 | 0.0315618 | 0.689968 |
| **5** | rs10773176 | G | A | -0.0172009 | 0.00228534 | 5.21E-14 | 3,176 | 409,005 | 0.0575803 | 0.0323954 | 0.0754988 |
| **6** | rs10805346 | C | T | 0.0117072 | 0.00202147 | 6.98E-09 | 3,176 | 409,005 | 0.0267622 | 0.0255965 | 0.295774 |
| **7** | rs10840270 | G | C | 0.014383 | 0.00212494 | 1.30E-11 | 3,176 | 409,005 | -0.0195029 | 0.0270101 | 0.470259 |
| **8** | rs10845387 | A | G | -0.0141214 | 0.00209396 | 1.54E-11 | 3,176 | 409,005 | 0.011475 | 0.0272305 | 0.673462 |
| **9** | rs10905255 | T | G | -0.0182493 | 0.00203099 | 2.58E-19 | 3,176 | 409,005 | 0.0412419 | 0.0256418 | 0.10775 |
| **10** | rs10977183 | T | C | -0.0136963 | 0.00206831 | 3.54E-11 | 3,176 | 409,005 | 0.0297762 | 0.0258893 | 0.250089 |
| **11** | rs1105407 | G | C | 0.0162208 | 0.0027778 | 5.24E-09 | 3,176 | 409,005 | 0.0532913 | 0.0333378 | 0.109926 |
| **12** | rs11085072 | T | C | -0.0131806 | 0.00236713 | 2.57E-08 | 3,176 | 409,005 | 0.039315 | 0.0345381 | 0.254992 |
| **13** | rs11117354 | C | T | 0.0232506 | 0.00219601 | 3.40E-26 | 3,176 | 409,005 | -0.0312901 | 0.0257275 | 0.223904 |
| **14** | rs111527438 | C | T | 0.0125 | 0.00211016 | 3.15E-09 | 3,176 | 409,005 | 0.0538227 | 0.0271069 | 0.0470804 |
| **15** | rs112037038 | G | A | -0.0167727 | 0.00200682 | 6.39E-17 | 3,176 | 409,005 | -0.0321884 | 0.0253952 | 0.204977 |
| **16** | rs112394943 | C | T | -0.0198961 | 0.00281641 | 1.61E-12 | 3,176 | 409,005 | -0.0442972 | 0.0303784 | 0.14479 |
| **17** | rs112511042 | C | T | -0.0351434 | 0.00416402 | 3.18E-17 | 3,176 | 409,005 | -0.0173426 | 0.0472919 | 0.713832 |
| **18** | rs113119217 | A | T | -0.0332464 | 0.00234274 | 1.04E-45 | 3,176 | 409,005 | -0.0265742 | 0.0297754 | 0.372131 |
| **19** | rs113525195 | A | C | -0.0124075 | 0.00224132 | 3.10E-08 | 3,176 | 409,005 | 0.00557643 | 0.0276744 | 0.840306 |
| **20** | rs11557154 | T | C | -0.0343719 | 0.00298538 | 1.13E-30 | 3,176 | 409,005 | -0.0188313 | 0.0375505 | 0.616023 |
| **21** | rs11579626 | C | A | 0.0265113 | 0.00357752 | 1.26E-13 | 3,176 | 409,005 | 0.0309795 | 0.0391235 | 0.428455 |
| **22** | rs11584821 | T | C | -0.0306517 | 0.00263623 | 3.00E-31 | 3,176 | 409,005 | 0.0180752 | 0.0330802 | 0.584787 |
| **23** | rs116863223 | A | G | -0.0817874 | 0.00937157 | 2.61E-18 | 3,176 | 409,005 | -0.114234 | 0.102079 | 0.263112 |
| **24** | rs11699829 | A | G | 0.0641957 | 0.00602028 | 1.51E-26 | 3,176 | 409,005 | 0.00724905 | 0.102986 | 0.943884 |
| **25** | rs117034449 | A | G | 0.0374377 | 0.00667898 | 2.08E-08 | 3,176 | 409,005 | 0.0917825 | 0.103623 | 0.37576 |
| **26** | rs117407747 | T | C | 0.0450533 | 0.00611706 | 1.77E-13 | 3,176 | 409,005 | -0.00463708 | 0.124438 | 0.970274 |
| **27** | rs117512405 | A | G | -0.0790134 | 0.00824611 | 9.53E-22 | 3,176 | 409,005 | -0.0502472 | 0.0569361 | 0.377497 |
| **28** | rs117630647 | A | G | 0.059565 | 0.00720413 | 1.36E-16 | 3,176 | 409,005 | 0.113405 | 0.0992225 | 0.253064 |
| **29** | rs11769630 | A | T | -0.0256807 | 0.00389475 | 4.29E-11 | 3,176 | 409,005 | -0.0166775 | 0.0418873 | 0.69052 |
| **30** | rs11866592 | A | G | 0.03464 | 0.00286611 | 1.25E-33 | 3,176 | 409,005 | 0.00962902 | 0.0324149 | 0.766424 |
| **31** | rs11991877 | A | T | -0.030138 | 0.00318686 | 3.17E-21 | 3,176 | 409,005 | -0.0530628 | 0.0363459 | 0.144308 |
| **32** | rs12369950 | C | T | -0.0178308 | 0.00290205 | 8.04E-10 | 3,176 | 409,005 | -0.0741315 | 0.03578 | 0.0382781 |
| **33** | rs12412214 | A | G | -0.0245174 | 0.00222685 | 3.42E-28 | 3,176 | 409,005 | 0.017752 | 0.026018 | 0.495051 |
| **34** | rs12451892 | C | T | -0.0116145 | 0.00207578 | 2.20E-08 | 3,176 | 409,005 | 0.00696711 | 0.0257532 | 0.78675 |
| **35** | rs12619538 | C | A | 0.0167489 | 0.00287813 | 5.91E-09 | 3,176 | 409,005 | -0.0418244 | 0.0365099 | 0.251975 |
| **36** | rs12925933 | C | A | -0.0146622 | 0.00213796 | 6.98E-12 | 3,176 | 409,005 | 0.0399901 | 0.0255386 | 0.117379 |
| **37** | rs12932179 | G | A | -0.0136257 | 0.0020276 | 1.82E-11 | 3,176 | 409,005 | 0.0507393 | 0.0260337 | 0.0512968 |
| **38** | rs13062095 | C | T | 0.0138552 | 0.00214113 | 9.74E-11 | 3,176 | 409,005 | 0.0242983 | 0.0262247 | 0.354164 |
| **39** | rs131794 | C | A | -0.0247859 | 0.00246292 | 8.00E-24 | 3,176 | 409,005 | 0.0140851 | 0.0326789 | 0.666457 |
| **40** | rs13230646 | C | T | -0.0173277 | 0.00232377 | 8.87E-14 | 3,176 | 409,005 | 0.0317927 | 0.0332864 | 0.339513 |
| **41** | rs1332941 | G | A | 0.0256552 | 0.00273159 | 5.88E-21 | 3,176 | 409,005 | -0.0217257 | 0.031345 | 0.488237 |
| **42** | rs137901416 | A | G | 0.04572 | 0.00332355 | 4.66E-43 | 3,176 | 409,005 | -0.0394482 | 0.0507418 | 0.436905 |
| **43** | rs139669835 | T | C | -0.0612563 | 0.0105346 | 6.07E-09 | 3,176 | 409,005 | -0.677237 | 0.355961 | 0.0570979 |
| **44** | rs139795227 | C | A | 0.0599379 | 0.00873247 | 6.71E-12 | 3,176 | 409,005 | -0.0214493 | 0.0797337 | 0.78792 |
| **45** | rs142426306 | T | C | -0.0504903 | 0.00539933 | 8.66E-21 | 3,176 | 409,005 | 0.0243393 | 0.0639385 | 0.70345 |
| **46** | rs143190905 | T | G | -0.0723995 | 0.00369421 | 1.61E-85 | 3,176 | 409,005 | 0.00850065 | 0.0484157 | 0.860627 |
| **47** | rs144204502 | T | C | -0.100574 | 0.00913369 | 3.37E-28 | 3,176 | 409,005 | 0.118153 | 0.067266 | 0.0790023 |
| **48** | rs150150565 | T | C | 0.063762 | 0.00739877 | 6.82E-18 | 3,176 | 409,005 | -0.159718 | 0.0951746 | 0.0933168 |
| **49** | rs17445108 | A | G | -0.0168922 | 0.00300983 | 2.00E-08 | 3,176 | 409,005 | -0.0250744 | 0.0384592 | 0.514418 |
| **50** | rs17677991 | G | C | 0.0222664 | 0.00210806 | 4.45E-26 | 3,176 | 409,005 | -0.000852754 | 0.0259039 | 0.973738 |
| **51** | rs182059586 | C | T | -0.0571159 | 0.00680853 | 4.91E-17 | 3,176 | 409,005 | -0.0452853 | 0.151017 | 0.764277 |
| **52** | rs185174247 | A | G | 0.0372806 | 0.00435145 | 1.06E-17 | 3,176 | 409,005 | -0.0215278 | 0.0730206 | 0.768134 |
| **53** | rs188918174 | T | C | 0.0403062 | 0.00543604 | 1.22E-13 | 3,176 | 409,005 | 0.00326097 | 0.0604453 | 0.956976 |
| **54** | rs1907702 | A | G | 0.0150247 | 0.00242651 | 5.94E-10 | 3,176 | 409,005 | 0.0370955 | 0.0318416 | 0.244019 |
| **55** | rs1957937 | T | A | 0.0209365 | 0.00273361 | 1.88E-14 | 3,176 | 409,005 | -0.0247202 | 0.0326557 | 0.449053 |
| **56** | rs1985369 | G | A | -0.0311893 | 0.00300952 | 3.63E-25 | 3,176 | 409,005 | 0.0415822 | 0.0411753 | 0.312552 |
| **57** | rs2056726 | A | G | -0.0228078 | 0.00243638 | 7.87E-21 | 3,176 | 409,005 | -0.0536192 | 0.0304561 | 0.0783159 |
| **58** | rs2282764 | G | A | -0.0224234 | 0.00289392 | 9.30E-15 | 3,176 | 409,005 | 0.0811342 | 0.036425 | 0.0259185 |
| **59** | rs2293579 | A | G | -0.012915 | 0.00205481 | 3.27E-10 | 3,176 | 409,005 | 0.00427932 | 0.0276636 | 0.877065 |
| **60** | rs2555104 | C | A | -0.0139717 | 0.00203498 | 6.61E-12 | 3,176 | 409,005 | 0.0350547 | 0.0253531 | 0.16677 |
| **61** | rs28363070 | A | G | 0.0755557 | 0.00959987 | 3.53E-15 | 3,176 | 409,005 | -0.342611 | 0.29213 | 0.240874 |
| **62** | rs28481848 | G | A | -0.0299462 | 0.00285068 | 8.20E-26 | 3,176 | 409,005 | 0.0385199 | 0.0462494 | 0.404915 |
| **63** | rs28502153 | A | C | -0.0215916 | 0.00206208 | 1.18E-25 | 3,176 | 409,005 | 0.0139993 | 0.0261603 | 0.592557 |
| **64** | rs28577594 | C | G | 0.0187657 | 0.00224024 | 5.45E-17 | 3,176 | 409,005 | 0.00491276 | 0.0280537 | 0.860986 |
| **65** | rs2967355 | C | A | -0.0461595 | 0.00238972 | 3.95E-83 | 3,176 | 409,005 | 0.0308449 | 0.0332103 | 0.353007 |
| **66** | rs2977608 | C | A | 0.0129483 | 0.00233716 | 3.02E-08 | 3,176 | 409,005 | -0.0127321 | 0.0275223 | 0.643643 |
| **67** | rs3093888 | A | G | -0.028973 | 0.00452459 | 1.52E-10 | 3,176 | 409,005 | 0.00158002 | 0.0448192 | 0.971878 |
| **68** | rs35640778 | A | G | -0.209011 | 0.00702087 | 9.57E-195 | 3,176 | 409,005 | 0.18467 | 0.114979 | 0.108249 |
| **69** | rs3767952 | A | G | 0.0134472 | 0.00238826 | 1.80E-08 | 3,176 | 409,005 | -0.0152693 | 0.0303958 | 0.615423 |
| **70** | rs3785074 | G | A | 0.023863 | 0.00220455 | 2.64E-27 | 3,176 | 409,005 | 0.024397 | 0.0310879 | 0.432585 |
| **71** | rs38664 | C | T | -0.0122471 | 0.00206433 | 2.98E-09 | 3,176 | 409,005 | -0.000447828 | 0.0254824 | 0.985979 |
| **72** | rs3891167 | G | A | -0.0425685 | 0.00239551 | 1.20E-70 | 3,176 | 409,005 | -0.00758452 | 0.0293853 | 0.796325 |
| **73** | rs41269079 | A | T | 0.0153617 | 0.0025499 | 1.70E-09 | 3,176 | 409,005 | -0.0459559 | 0.0311334 | 0.139918 |
| **74** | rs41304832 | A | G | 0.0611702 | 0.0093095 | 5.01E-11 | 3,176 | 409,005 | -0.0306881 | 0.0667427 | 0.645662 |
| **75** | rs429358 | C | T | 0.0173498 | 0.00277091 | 3.82E-10 | 3,176 | 409,005 | -0.0322457 | 0.0330233 | 0.32884 |
| **76** | rs4498805 | T | G | 0.0150601 | 0.00200376 | 5.65E-14 | 3,176 | 409,005 | -0.0325457 | 0.0253246 | 0.198743 |
| **77** | rs4530278 | T | G | 0.0138793 | 0.0020567 | 1.50E-11 | 3,176 | 409,005 | -0.00138775 | 0.0259314 | 0.957321 |
| **78** | rs4695407 | G | A | 0.0141511 | 0.00199925 | 1.46E-12 | 3,176 | 409,005 | -0.00165623 | 0.0254195 | 0.94805 |
| **79** | rs4724 | A | G | -0.0547446 | 0.00312441 | 9.81E-69 | 3,176 | 409,005 | 0.0754737 | 0.0408203 | 0.0644689 |
| **80** | rs4743037 | T | C | 0.0147971 | 0.00238094 | 5.14E-10 | 3,176 | 409,005 | -0.0108247 | 0.030728 | 0.724631 |
| **81** | rs55747751 | A | G | -0.0211612 | 0.00375164 | 1.70E-08 | 3,176 | 409,005 | 0.0921141 | 0.0547834 | 0.0926808 |
| **82** | rs56799554 | G | A | -0.0259793 | 0.00267858 | 3.05E-22 | 3,176 | 409,005 | 0.0066717 | 0.0302252 | 0.8253 |
| **83** | rs5742915 | C | T | 0.0193377 | 0.00202886 | 1.55E-21 | 3,176 | 409,005 | -0.0109191 | 0.0257692 | 0.671766 |
| **84** | rs59409453 | G | A | 0.0202133 | 0.00230175 | 1.61E-18 | 3,176 | 409,005 | -0.0143776 | 0.0314317 | 0.647368 |
| **85** | rs6007020 | C | T | 0.0144904 | 0.00209637 | 4.77E-12 | 3,176 | 409,005 | -0.0269768 | 0.0265129 | 0.308916 |
| **86** | rs6054257 | A | G | -0.0141684 | 0.00247729 | 1.07E-08 | 3,176 | 409,005 | 0.0150245 | 0.031954 | 0.638216 |
| **87** | rs611646 | A | T | -0.0368309 | 0.00203547 | 3.52E-73 | 3,176 | 409,005 | 0.00178231 | 0.0259081 | 0.945154 |
| **88** | rs61748181 | T | C | -0.059181 | 0.00595394 | 2.79E-23 | 3,176 | 409,005 | 0.0122335 | 0.0566745 | 0.8291 |
| **89** | rs6536702 | A | G | 0.0534148 | 0.00238875 | 9.44E-111 | 3,176 | 409,005 | 0.00960938 | 0.0325991 | 0.768166 |
| **90** | rs6584579 | G | A | 0.0114923 | 0.00204674 | 1.97E-08 | 3,176 | 409,005 | 0.00909193 | 0.0270732 | 0.737001 |
| **91** | rs6587577 | G | A | -0.0182148 | 0.0026359 | 4.84E-12 | 3,176 | 409,005 | -0.00785476 | 0.0346236 | 0.820531 |
| **92** | rs6659669 | T | C | -0.0117091 | 0.00205167 | 1.15E-08 | 3,176 | 409,005 | 0.0542737 | 0.0257021 | 0.0347168 |
| **93** | rs6669563 | A | G | 0.0182358 | 0.00202476 | 2.13E-19 | 3,176 | 409,005 | -0.0201234 | 0.0254633 | 0.429359 |
| **94** | rs66731853 | A | G | -0.0177791 | 0.00215421 | 1.54E-16 | 3,176 | 409,005 | 0.00251618 | 0.0320329 | 0.937391 |
| **95** | rs6751209 | C | T | -0.0140465 | 0.00248465 | 1.57E-08 | 3,176 | 409,005 | -0.00835803 | 0.0328715 | 0.799292 |
| **96** | rs6776756 | A | G | -0.0174439 | 0.00203747 | 1.11E-17 | 3,176 | 409,005 | -0.043379 | 0.0256656 | 0.0909976 |
| **97** | rs6873104 | T | A | -0.0245329 | 0.00335421 | 2.59E-13 | 3,176 | 409,005 | 0.0573621 | 0.040868 | 0.16044 |
| **98** | rs6881568 | A | C | 0.0169256 | 0.00207735 | 3.71E-16 | 3,176 | 409,005 | 0.00149356 | 0.0284387 | 0.958116 |
| **99** | rs7099229 | A | G | -0.0153288 | 0.00224403 | 8.44E-12 | 3,176 | 409,005 | 0.0246428 | 0.0331625 | 0.457426 |
| **100** | rs7164950 | G | A | 0.0129362 | 0.00204001 | 2.28E-10 | 3,176 | 409,005 | 0.0129701 | 0.0257934 | 0.615072 |
| **101** | rs7209057 | A | G | 0.011819 | 0.00202865 | 5.68E-09 | 3,176 | 409,005 | 0.00239803 | 0.0255395 | 0.925193 |
| **102** | rs7221585 | T | C | 0.0143271 | 0.00247042 | 6.65E-09 | 3,176 | 409,005 | 0.011635 | 0.0282053 | 0.679966 |
| **103** | rs73581419 | T | C | 0.0229838 | 0.00324156 | 1.34E-12 | 3,176 | 409,005 | 0.00604326 | 0.0462821 | 0.896112 |
| **104** | rs73730598 | A | G | 0.0273632 | 0.00439272 | 4.69E-10 | 3,176 | 409,005 | -0.0300471 | 0.05101 | 0.555833 |
| **105** | rs7555872 | G | A | 0.027267 | 0.00498734 | 4.57E-08 | 3,176 | 409,005 | 0.0131474 | 0.0656912 | 0.841372 |
| **106** | rs76219171 | A | G | 0.0359839 | 0.00431741 | 7.78E-17 | 3,176 | 409,005 | 0.00510182 | 0.0691169 | 0.941158 |
| **107** | rs762679 | A | T | 0.0310104 | 0.00285024 | 1.44E-27 | 3,176 | 409,005 | -0.0622424 | 0.0335971 | 0.0639381 |
| **108** | rs762810 | A | C | -0.0202965 | 0.00210014 | 4.27E-22 | 3,176 | 409,005 | 0.0167048 | 0.0259285 | 0.519405 |
| **109** | rs76666449 | C | T | 0.0295125 | 0.00333186 | 8.17E-19 | 3,176 | 409,005 | 0.0527352 | 0.0368014 | 0.151868 |
| **110** | rs77231040 | C | G | 0.0989303 | 0.0134649 | 2.02E-13 | 3,176 | 409,005 | -0.0691498 | 0.0793744 | 0.383652 |
| **111** | rs7772289 | T | G | 0.017549 | 0.00200003 | 1.72E-18 | 3,176 | 409,005 | -0.0925463 | 0.0257484 | 0.000325319 |
| **112** | rs7790856 | T | C | -0.0437199 | 0.00220526 | 1.80E-87 | 3,176 | 409,005 | 0.0261331 | 0.030332 | 0.388925 |
| **113** | rs78491606 | C | A | -0.0756311 | 0.00741168 | 1.90E-24 | 3,176 | 409,005 | 0.0497614 | 0.0769311 | 0.517742 |
| **114** | rs79755767 | A | G | 0.0278665 | 0.00343286 | 4.76E-16 | 3,176 | 409,005 | -0.00318017 | 0.0427218 | 0.940661 |
| **115** | rs8006485 | T | G | 0.0191453 | 0.00200238 | 1.16E-21 | 3,176 | 409,005 | 0.00269459 | 0.0260492 | 0.917612 |
| **116** | rs80324517 | A | G | 0.0396515 | 0.00466286 | 1.84E-17 | 3,176 | 409,005 | -0.0124213 | 0.0523789 | 0.812546 |
| **117** | rs8102497 | A | G | -0.0149654 | 0.0020233 | 1.40E-13 | 3,176 | 409,005 | 0.0100256 | 0.0255062 | 0.694271 |
| **118** | rs8105767 | G | A | 0.0328384 | 0.00220117 | 2.49E-50 | 3,176 | 409,005 | 0.00426911 | 0.0272654 | 0.875579 |
| **119** | rs869785 | C | T | -0.0147303 | 0.00212801 | 4.45E-12 | 3,176 | 409,005 | 0.0559635 | 0.026827 | 0.0369709 |
| **120** | rs871134 | T | C | -0.0182986 | 0.0020263 | 1.71E-19 | 3,176 | 409,005 | -0.0378408 | 0.0255017 | 0.137847 |
| **121** | rs932002 | T | C | -0.0402052 | 0.00279667 | 7.31E-47 | 3,176 | 409,005 | 0.0275251 | 0.0297898 | 0.355498 |
| **122** | rs9398196 | G | A | -0.0143586 | 0.00201175 | 9.51E-13 | 3,176 | 409,005 | -0.0155065 | 0.0261108 | 0.552598 |
| **123** | rs939916 | A | G | 0.0241795 | 0.00216724 | 6.63E-29 | 3,176 | 409,005 | -0.000886246 | 0.0287347 | 0.975395 |
| **124** | rs9419958 | C | T | -0.0810098 | 0.00293847 | 2.64E-167 | 3,176 | 409,005 | 0.0302077 | 0.0394169 | 0.44346 |
| **125** | rs9600019 | T | C | 0.0127134 | 0.00213096 | 2.43E-09 | 3,176 | 409,005 | 0.0259967 | 0.0262815 | 0.322583 |
| **126** | rs965109 | T | C | -0.101702 | 0.00648201 | 1.77E-55 | 3,176 | 409,005 | -0.0477003 | 0.0490146 | 0.330462 |
| **127** | rs9878436 | T | C | -0.0143407 | 0.00201819 | 1.20E-12 | 3,176 | 409,005 | -0.0261082 | 0.0265168 | 0.324825 |
| **128** | rs9955360 | A | C | -0.0190311 | 0.00299791 | 2.18E-10 | 3,176 | 409,005 | -0.0536063 | 0.0362233 | 0.138905 |

SNP, Single Nucleotide Polymorphism; EA, Effect Allele; OA, Other Allele; SE, Standard Error; TL, Telomere Length; GD, Graves' Disease.

**Supplementary Table S4** The results of MR-Egger intercept analysis.

| **Exposure** | **Outcome** | **Egger_intercept** | **SE** | **p-value** |
| --- | --- | --- | --- | --- |
| TL | AIT | -0.008563206 | 0.013241473 | 0.519005104 |
| TL | GD | -0.002145278 | 0.005463486 | 0.695236049 |

TL, Telomere Length; AIT, Autoimmune Thyroiditis; GD, Graves' Disease.

**Supplementary Table S5** The results of heterogeneity analysis.

| **Exposure** | **Outcome** | **Method** | ***Q*** | ***Q*_df** | ***Q*_p val** |
| --- | --- | --- | --- | --- | --- |
| TL | AIT | MR Egger | 133.5681589 | 126 | 0.30526013 |
| TL | AIT | Inverse variance weighted | 134.0114944 | 127 | 0.317838212 |
| TL | GD | MR Egger | 131.7018978 | 126 | 0.346140145 |
| TL | GD | Inverse variance weighted | 131.8630547 | 127 | 0.365784316 |

TL, Telomere Length; AIT, Autoimmune Thyroiditis; GD, Graves' Disease.
